# Impact of upper and lower respiratory symptoms on COVID-19 outcomes: a multicenter retrospective cohort study

**DOI:** 10.1186/s12931-022-02222-3

**Published:** 2022-11-15

**Authors:** Kensuke Nakagawara, Shotaro Chubachi, Ho Namkoong, Hiromu Tanaka, Ho Lee, Shuhei Azekawa, Shiro Otake, Takahiro Fukushima, Atsuho Morita, Mayuko Watase, Kaori Sakurai, Tatsuya Kusumoto, Takanori Asakura, Katsunori Masaki, Hirofumi Kamata, Makoto Ishii, Naoki Hasegawa, Norihiro Harada, Tetsuya Ueda, Soichiro Ueda, Takashi Ishiguro, Ken Arimura, Fukuki Saito, Takashi Yoshiyama, Yasushi Nakano, Yoshikazu Mutoh, Yusuke Suzuki, Ryuya Edahiro, Koji Murakami, Yasunori Sato, Yukinori Okada, Ryuji Koike, Yuko Kitagawa, Katsushi Tokunaga, Akinori Kimura, Seiya Imoto, Satoru Miyano, Seishi Ogawa, Takanori Kanai, Koichi Fukunaga

**Affiliations:** 1 Division of Pulmonary Medicine, Department of Medicine, Keio University School of Medicine, 35 Shinanomachi, Shinjuku-Ku, Tokyo, 160-8582 Japan; 2 Department of Infectious Diseases, Keio University School of Medicine, 35 Shinanomachi, Shinjuku-Ku, Tokyo, 160-8582 Japan; 3 Department of Clinical Medicine (Laboratory of Bioregulatory Medicine), Kitasato University School of Pharmacy, Tokyo, Japan; 4 Department of Respiratory Medicine, Kitasato University, Kitasato Institute Hospital, Tokyo, Japan; 5 Department of Respiratory Medicine, Nagoya University Graduate School of Medicine, Nagoya, Japan; 6 Department of Respiratory Medicine, Juntendo University Faculty of Medicine and Graduate School of Medicine, Tokyo, Japan; 7 Department of Respiratory Medicine, Osaka Saiseikai Nakatsu Hospital, Osaka, Japan; 8 Department of Internal Medicine, JCHO (Japan Community Health Care Organization) Saitama Medical Center, Saitama, Japan; 9 Department of Respiratory Medicine, Saitama Cardiovascular and Respiratory Center, Kumagaya, Japan; 10 Department of Respiratory Medicine, Tokyo Women’s Medical University, Tokyo, Japan; 11 Department of Emergency and Critical Care Medicine, Kansai Medical University General Medical Center, Moriguchi, Japan; 12 Respiratory Disease Center, Fukujuji Hospital, Japan Anti-Tuberculosis Association, Tokyo, Japan; 13 Department of Internal Medicine, Kawasaki Municipal Ida Hospital, Kawasaki, Japan; 14 Department of Infectious Diseases, Tosei General Hospital, Seto, Japan; 15 Department of Respiratory Medicine and Clinical Immunology, Osaka University Graduate School of Medicine, Suita, Japan; 16 Department of Respiratory Medicine, Tohoku University Graduate School of Medicine, Sendai, Japan; 17 Department of Preventive Medicine and Public Health, Keio University School of Medicine, Tokyo, Japan; 18 Department of Statistical Genetics, Osaka University Graduate School of Medicine, Suita, Japan; 19 Integrated Frontier Research for Medical Science Division, Institute for Open and Transdisciplinary Research Initiatives, Osaka University, Suita, Japan; 20 The Center for Infectious Disease Education and Research (CiDER), Osaka University, Suita, Japan; 21 Laboratory of Statistical Immunology, Immunology Frontier Research Center (WPI-IFReC), Osaka University, Suita, Japan; 22 Department of Genome Informatics, Graduate School of Medicine, The University of Tokyo, Tokyo, Japan; 23 Laboratory for Systems Genetics, RIKEN Center for Integrative Medical Sciences, Kanagawa, Japan; 24 Medical Innovation Promotion Center, Tokyo Medical and Dental University, Tokyo, Japan; 25 Department of Surgery, Keio University School of Medicine, Tokyo, Japan; 26 Genome Medical Science Project (Toyama), National Center for Global Health and Medicine, Tokyo, Japan; 27 Institute of Research, Tokyo Medical and Dental University, Tokyo, Japan; 28 Division of Health Medical Intelligence, Human Genome Center, The Institute of Medical Science, the University of Tokyo, Tokyo, Japan; 29 M&D Data Science Center, Tokyo Medical and Dental University, Tokyo, Japan; 30 Department of Pathology and Tumor Biology, Kyoto University, Kyoto, Japan; 31 Institute for the Advanced Study of Human Biology (WPI-ASHBi), Kyoto University, Kyoto, Japan; 32 Department of Medicine, Center for Hematology and Regenerative Medicine, Karolinska Institute, Stockholm, Sweden; 33 Division of Gastroenterology and Hepatology, Department of Medicine, Keio University School of Medicine, Tokyo, Japan

**Keywords:** SARS-CoV-2 infection, COVID-19, Upper respiratory tract symptoms, Lower respiratory tract symptoms, Primary care

## Abstract

**Background:**

Respiratory symptoms are associated with coronavirus disease 2019 (COVID-19) outcomes. However, the impacts of upper and lower respiratory symptoms on COVID-19 outcomes in the same population have not been compared. The objective of this study was to characterize upper and lower respiratory symptoms and compare their impacts on outcomes of hospitalized COVID-19 patients.

**Methods:**

This was a multicenter, retrospective cohort study; the database from the Japan COVID-19 Task Force was used. A total of 3314 COVID-19 patients were included in the study, and the data on respiratory symptoms were collected. The participants were classified according to their respiratory symptoms (Group 1: no respiratory symptoms, Group 2: only upper respiratory symptoms, Group 3: only lower respiratory symptoms, and Group 4: both upper and lower respiratory symptoms). The impacts of upper and lower respiratory symptoms on the clinical outcomes were compared. The primary outcome was the percentage of patients with poor clinical outcomes, including the need for oxygen supplementation via high-flow oxygen therapy, mechanical ventilation, and extracorporeal membrane oxygenation or death.

**Results:**

Of the 3314 COVID-19 patients, 605, 1331, 1229, and 1149 were classified as Group 1, Group 2, Group 3, and Group 4, respectively. In univariate analysis, patients in Group 2 had the best clinical outcomes among all groups (odds ratio [OR]: 0.21, 95% confidence interval [CI]: 0.11–0.39), while patients in Group 3 had the worst outcomes (OR: 3.27, 95% CI: 2.43–4.40). Group 3 patients had the highest incidence of pneumonia, other complications due to secondary infections, and thrombosis during the clinical course.

**Conclusions:**

Upper and lower respiratory tract symptoms had vastly different impacts on the clinical outcomes of COVID-19.

**Supplementary Information:**

The online version contains supplementary material available at 10.1186/s12931-022-02222-3.

## Background

The most common symptoms of coronavirus disease 2019 (COVID-19) are cough, myalgia, and headache [1]. Additionally, various symptoms including gastrointestinal symptoms (diarrhea), dysgeusia, and dysosmia have been reported in COVID-19 patients [2, 3]. Of the 1.3 million patients reported by the Centers for Disease Control and Prevention (CDC) at the end of May 2020, 14% were hospitalized, 2% were treated in the intensive care unit (ICU), and 5% died [2, 4]. In recent years, several predictive tools have been proposed and used to identify patients prone to severe disease based on epidemiological, clinical, and laboratory characteristics [5, 6]. Primary physicians need to identify patients prone to severe outcomes based on limited clinical information and direct them to the appropriate higher-level medical facilities. Data on respiratory symptoms can be easily obtained during patient visits and could be crucial for primary care physicians.

Upper respiratory symptoms were reported to be present more frequently in COVID-19 than in the influenza virus infection [7, 8]. While sore throat and nasal discharge were reported in approximately 14.4% and 7.7% of the cases [9], respectively, dysgeusia or dysosmia were observed in 62% of the cases and were considered typical upper respiratory symptoms [8, 10]. Angiotensin-converting enzyme 2 (ACE2) receptors are highly expressed in the nasal epithelium, acting as entry and replication points for severe acute respiratory syndrome coronavirus 2 (SARS-CoV-2) [11], causing dysfunction of the olfactory neurons and taste buds and resulting in dysgeusia or dysosmia [12], although the exact mechanism is still unknown [13]. Additionally, dysosmia and dysgeusia were associated with the medical history of COVID-19 patients [12–15], with a higher incidence in younger adults and women with no comorbidities [12]. Dysosmia and dysgeusia occur in more than half of COVID-19 patients [8, 10]; however, previous studies have revealed an incidence of 4% in hospitalized patients [12, 16]. Thus, there could be an inverse association between dysosmia/dysgeusia and favorable clinical outcomes [13–15].

In the context of lower respiratory symptoms, a systematic review of 152 previous studies suggested cough as the most common symptom of COVID-19, occurring in approximately 50% of the cases [9]. Other lower respiratory symptoms such as sputum production and dyspnea were observed in approximately 25–30% of the cases [9]. Lower respiratory symptoms of cough and dyspnea indicate pneumonia and are associated with severe clinical outcomes [2, 6, 17, 18]. Additionally, some studies have suggested that cough and sputum production during the clinical course were caused by secondary bacterial infections [19, 20].

Hence, we hypothesized that these respiratory symptoms could be related to the clinical outcomes. However, no reports have compared the effect of both upper and lower respiratory symptoms on clinical outcomes. The aim of this present study was to investigate the impact of respiratory symptoms on the clinical outcomes of patients hospitalized with COVID-19.

## Methods

### Study design and settings

In this retrospective cohort study, data were collected from the Japan COVID-19 Task Force database from February 2020 to November 2021. The Japan COVID-19 Task Force collected clinical information on patients with COVID-19 aged > 18 years and diagnosed by polymerase chain reaction test or antigen test from 78 hospitals nationwide in Japan [21, 22]. Of the 3431 patients identified, 117 patients were excluded due to unknown respiratory symptoms, and thus, 3314 patients were included in the analysis (Additional file 1: Fig. S1). This study was approved by the Ethics Committee of Keio University School of Medicine (ID: 20200061), and written or oral informed consent was obtained. The study was conducted in accordance with the 1964 Declaration of Helsinki and its later amendments.

### Definition of respiratory symptoms

Sore throat, nasal discharge, dysosmia, and dysgeusia were categorized as upper respiratory symptoms, while cough, sputum production, and dyspnea were categorized as lower respiratory symptoms. Based on the presence of upper or lower respiratory symptoms, the enrolled patients were classified into four groups as follows: Group 1: patients with no respiratory symptoms at all during the clinical course; Group 2: patients with only upper respiratory symptoms; Group 3: patients with only lower respiratory symptoms; and Group 4: patients with both upper and lower respiratory symptoms. The presence of all symptoms was reported subjectively by the patients, and the corresponding data were collected by the health care provider through medical interviews.

### Data collection

The following patient data were obtained from the electronic case record form: age, sex, body mass index, number of days in the hospital, comorbidities, clinical symptoms and signs, laboratory and radiographic findings, complications after hospitalization, and medications administered during hospital stay (remdesivir, antibiotics, steroids, tocilizumab, baricitinib, and anti-coagulant drugs). In this study, poor clinical outcomes were defined as the need for oxygen supplementation via high-flow oxygen therapy, mechanical ventilation, and extracorporeal membrane oxygenation (ECMO) or death [22, 23]. All laboratory tests and radiography were performed within 48 h of the initial visit or admission based on the clinical care needs of the patients. The primary outcome was the percentage of patients with poor clinical outcomes.

### Statistical analysis

For baseline variables, we reported categorial variables as frequencies and proportions and continuous variables as mean and standard error. Data were compared among the four groups using the chi-square test, ANOVA, and Dunnett’s test. In Dunnett’s tests, Group 1 was used as a control and was compared with the other groups. To assess the association between respiratory symptoms and poor clinical outcomes, we performed univariate analysis and calculated the odds ratio (OR). Data are presented as OR with 95% confidence interval (95% CI). Statistical significance was set at *p* < 0.05. To investigate the relationship between each group and poor prognosis, we performed a multivariable logistic regression analysis to adjust for previously reported factors [24–30]. Specifically, the models were adjusted for patient characteristics, such as age, sex, body mass index (BMI), smoking history, and comorbidities (hypertension, diabetes, cardiovascular disease and chronic kidney disease). We presented the adjusted odds ratio (aOR) with a 95% CI. Statistical significance was set at p < 0.05. All data were analyzed using the JMP 16 program (SAS Institute Japan Ltd., Tokyo, Japan).

## Results

### Comparison of baseline characteristics between the four groups stratified by respiratory symptoms

Table 1 shows the clinical characteristics of each group. Among the 3314 COVID-19 patients, 605 patients had no respiratory symptoms (Group 1). There were 2709 COVID-19 patients with respiratory symptoms, including 331 patients with only upper respiratory symptoms (Group 2), 1229 patients with only lower respiratory symptoms (Group 3), and 1149 patients with both upper and lower respiratory symptoms (Group 4). On comparing the clinical characteristics of patients in the four groups, parameters such as age and the incidence of hypertension, diabetes, cardiovascular disorders, and chronic kidney disease, generally associated with the severity of COVID-19 [24–28], were significantly lower in Groups 2 and 4 than in Group 1 (*p* < 0.05). The proportion of males and patients with a higher BMI, considered factors associated with severe outcomes of COVID-19 [29, 30], was significantly higher in Group 3.

**Table 1:** Main clinical characteristics of each group

	All (n = 3314)	Group 1 (n = 605)	Group 2 (n = 331)	Group 3 (n = 1229)	Group 4 (n = 1149)	*p* value
Age, years	56.5 ± 17.5	62.0 ± 18.6	48.2 ± 19.2	60.2 ± 15.7	52.1 ± 16.3	< 0.0001^a = **/c = **^
Sex (Male), %	67	65.5	59.8	71.3	65.4	0.0001 ^b= **^
BMI	24.8 ± 4.8	23.9 ± 4.9	23.5 ± 4.1	25.2 ± 4.9	25.2 ± 4.8	< 0.0001^b = **/c = **^
Days of onset	5.74 ± 4.0	4.35 ± 3.8	4.64 ± 3.3	6.29 ± 4.1	6.18 ± 4.0	< 0.0001^b = **/c = **^
Smoker, %	14.8	12.2	16.2	13.3	17.5	0.0090^c = **^
Hypertension, %	33.5	41.3	21	40.4	25.8	< 0.0001^a = **/c = **^
Diabetes, %	21	22.2	15.2	25.8	16.8	< 0.0001 ^a = **/c = **^
Cardiovascular disorders, %	10.2	13.7	4.9	13	6.9	< 0.0001 ^a = **/c = **^
COPD, %	4.1	3.9	3.1	5.7	2.8	0.0031
Chronic kidney disease, %	7	9.4	4.6	8.8	4.4	< 0.0001^a = */c = **^
Cancer, %	6.6	9.4	5.2	5.8	6.4	0.0175^a = */b = **/c = *^
Hyperuricemia, %	9.9	11.2	7.3	10.8	9.1	0.1456
Chronic liver disease, %	4.3	4.8	3.1	4.6	4.2	0.6131
Asthma, %	7.2	5.3	5.8	7.6	8.2	0.1056
Fever, %	80.7	72.3	71.5	81.7	86.7	< 0.0001^b = **/c = **^
WBC (/μL)	5771.8 ± 2873.8	5560.0 ± 2495.1	5371.8 ± 2604.4	6266.3 ± 3406.3	5466.4 ± 2399.6	< 0.0001^b = **^
Neutrophil (/μL)	4584.2 ± 10,509.2	3916.2 ± 2282.4	3648.2 ± 2204.4	5530.7 ± 1494.3	4190.8 ± 4190.8	0.0013^b = **^
Lymphocytes (/μL)	1145.2 ± 2342.1	1126.0 ± 556.0	1250.0 ± 595.8	1127.0 ± 3326.0	1145.3 ± 1920.1	0.8689
Neutrophil lymphocyte ratio	6.13 ± 17.0	4.85 ± 7.0	3.64 ± 3.6	7.27 ± 11.0	6.29 ± 25.9	0.0018 ^b = *^
Eosinophil (/μL)	42.3 ± 184.9	57.1 ± 153.5	58.2 ± 188.7	36.8 ± 258.9	36.1 ± 65.5	0.0429
AST (IU/L)	43.0 ± 58.5	38.9 ± 76.9	36.1 ± 98.5	47.1 ± 49.1	42.7 ± 37.3	0.0040^b = *^
ALT (IU/L)	39.6 ± 71.5	32.9 ± 38.2	39.9 ± 190.5	42.1 ± 45.9	40.4 ± 38.5	0.0755^b = *^
T-B (mg/dL)	0.7 ± 0.4	0.7 ± 0.4	0.7 ± 0.3	0.7 ± 0.5	0.6 ± 0.3	0.1603
γ-GTP (IU/L)	69.0 ± 87.5	55.7 ± 67.5	47.5 ± 63.1	75.7 ± 90.1	74.7 ± 97.5	< 0.0001^b = **/c = **^
Alb (mg/dL)	3.7 ± 0.6	3.8 ± 0.6	4.1 ± 0.5	3.5 ± 0.6	3.8 ± 0.6	< 0.0001^a = **/b = **^
BUN (mg/dL)	16.9 ± 11.8	18.1 ± 12.8	14.4 ± 8.8	18.9 ± 13.1	14.9 ± 9.9	< 0.0001^a = **/c = **^
Cr (mg/dL)	1.1 ± 1.3	1.1 ± 1.6	1.0 ± 1.7	1.1 ± 1.4	1.0 ± 1.0	0.0220^c = *^
LDH (IU/L)	292.2 ± 153.2	255.9 ± 131.1	222.6 ± 91.5	333.8 ± 176.0	286.3 ± 138.2	< 0.0001^a = **/b = **/c = **^
UA (mg/dL)	4.9 ± 1.8	5.2 ± 1.9	4.8 ± 1.7	4.9 ± 1.9	4.7 ± 1.6	0.0002^a = **/c = **^
HbA1c (%)	6.4 ± 1.3	6.3 ± 1.4	6.0 ± 1.0	6.6 ± 1.4	6.2 ± 1.2	< 0.0001^a = */b = **^
CRP (mg/dL)	5.7 ± 27.7	3.8 ± 5.1	2.7 ± 4.0	6.8 ± 6.9	6.4 ± 46.5	0.0286
Procalcitonin (ng/mL)	0.6 ± 17.2	0.2 ± 1.1	0.2 ± 0.8	1.4 ± 28.5	0.2 ± 0.6	0.3982
D-dimer (μg/mL)	2.2 ± 7.9	2.3 ± 8.4	1.2 ± 2.1	3.1 ± 11.0	1.4 ± 3.5	< 0.0001
Ferritin (ng/mL)	628.0 ± 760.1	518.0 ± 880.4	390.9 ± 497.7	758.1 ± 768.4	611.2 ± 722.5	< 0.0001^b = **^
BNP (pg/mL)	54.8 ± 287.4	55.5 ± 146.3	21.8 ± 51.3	89.1 ± 436.1	25.0 ± 91.4	0.0004
KL-6 (IU/L)	328.7 ± 326.3	300.9 ± 337.6	235.1 ± 116.2	393.5 ± 402.2	299.1 ± 249.1	< 0.0001^a = */b = **^

Data are shown as mean ± standard Deviation (SD)

*BMI* body mass index, *COPD* chronic obstructive pulmonary disease, *WBC* white blood cell, *AST* aspartate aminotransferase, *ALT* alanine aminotransferase, *T-B* total bilirubin, *Alb* albumin, *BUN* blood urea nitrogen, *Cr* creatinine, *LDH* lactate dehydrogenase, *UA* uric acid, *CRP* C-reactive protein, *BNP* brain natriuretic peptide, *KL-6* Krebs von den Lungen-6

^a^Comparison of patients in group 1 versus group 2

^b^Comparison of patients in group 1 versus group 3

^c^Comparison of patients in group 1 versus group 4

*p < 0.05 ** *p* < 0.01

### Laboratory results of the patients in the four groups

The clinical laboratory findings of the enrolled patients are presented in Table 1. Patients in Group 3 had higher levels of white blood cells, neutrophils, aspartate aminotransferase (AST), alanine aminotransferase (ALT), HbA1c, and ferritin; neutrophil lymphocyte ratio (NLR); and Krebs von den Lungen-6 values (all *p* < 0.05) than the patients in Group 1. Conversely, albumin (Alb), blood urea nitrogen, uric acid, HbA1c, and Krebs von den Lungen-6 levels (all *p* < 0.05) of Group 2 patients were significantly lower than those of Group 1 patients. The lactate dehydrogenase (LDH) levels in Group 2 patients were significantly lower than those of Group 1 patients, whereas LDH levels were significantly higher in Group 3 and 4 patients than in Group 1 patients.

### Upper and lower respiratory symptoms of the patients in the four groups

**Fig. 1:**
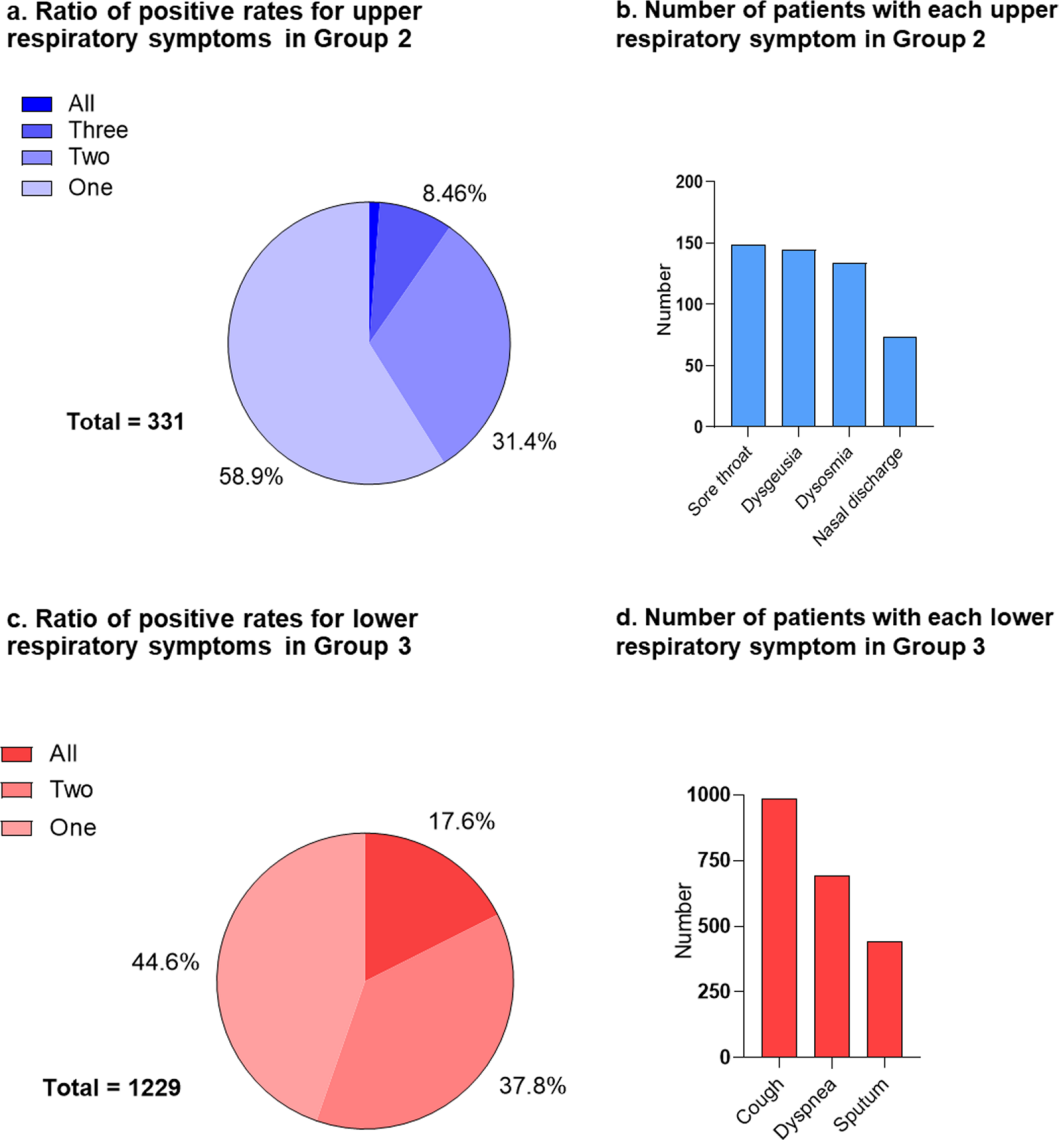
Details of upper and lower respiratory symptoms. **a** Ratio of upper respiratory symptoms in Group 2. Of 331 patients in Group 2, 136 patients (41.1%) developed two or more upper respiratory symptoms at the same time. **b** Number of patients with each upper respiratory symptom in Group 2. Of 331 patients in Group 2, 149 patients developed sore throat, the most frequent upper respiratory symptom. Dysosmia and dysgeusia were also as common as sore throat. **c** Ratio of lower respiratory symptoms in Group 3. Of 1229 patients in Group 3, 549 patients (44.6%) developed a single lower respiratory symptom. **d** Number of patients with each lower respiratory symptom in Group 3. Of 1229 patients in Group 3, 988 patients presented with cough and 695 patients with dyspnea. The frequency of sputum production was the lowest

In Group 2, most patients (58.9%) suffered from only one upper respiratory symptom. The frequency decreased as the number of symptoms increased, with only four patients (1.2%) developing all four upper respiratory symptoms (Fig. 1a). The most common upper respiratory symptom was sore throat (149 cases), followed by dysgeusia (145 cases) and dysosmia (134 cases). The incidence of nasal discharge was the lowest (74 cases) (Fig. 1b). The details of lower respiratory symptoms were as follows: 549 (44.6%) patients developed only one lower respiratory symptom, 464 (37.8%) developed two symptoms, and 216 (17.6%) patients developed all lower respiratory symptoms (Fig. 1c). Among these, cough was the most frequent symptom (988 cases), followed by dyspnea (695 cases) and sputum production (443 cases) (Fig. 1d). The most common symptoms in all groups, excluding respiratory presentations, were fever, fatigue, and diarrhea (Additional file 1: Table S1). In Group 4 patients, all systemic symptoms, except bloody stools, were significantly more frequently noted than in Group 1 patients. In contrast, only fever and fatigue were more prevalent in Group 3 patients.

### Radiographic findings of the patients in the four groups

**Fig. 2:**
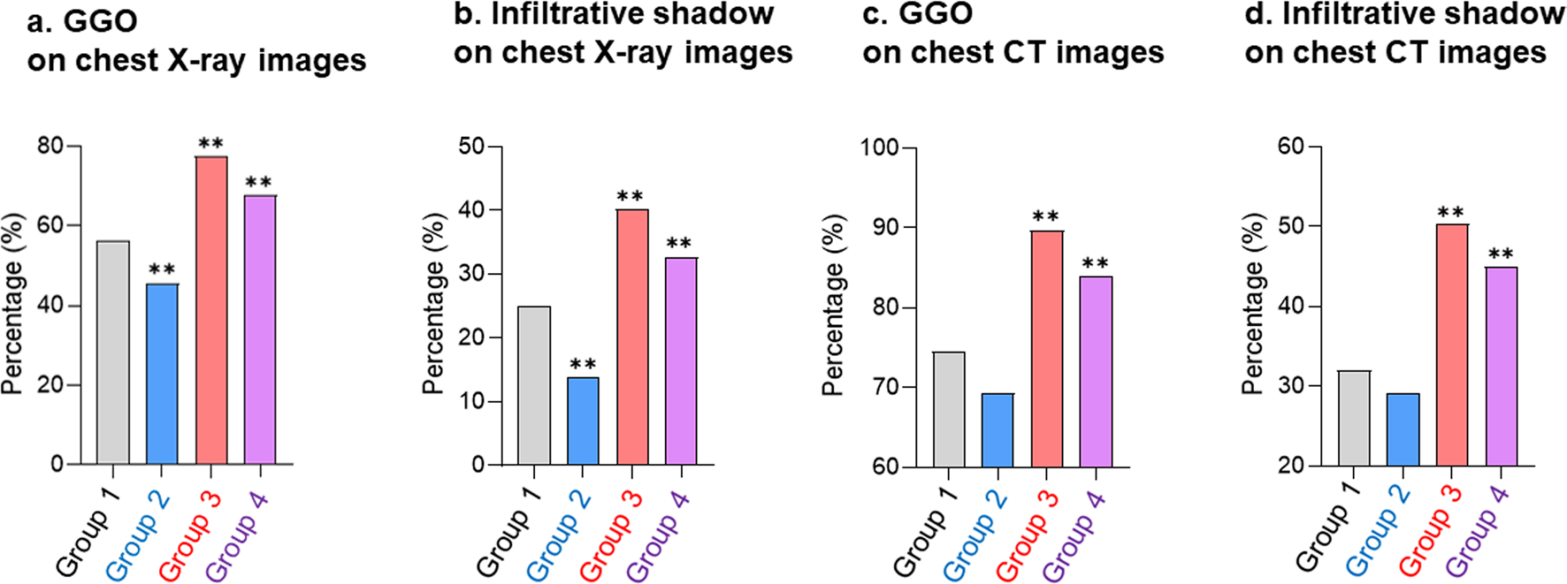
Comparison of chest images among the four groups. **a, b** Proportions of patients with ground-glass opacities and infiltrative shadows on chest X-ray images among the four groups. **c, d** Proportions of patients with ground-glass opacities and infiltrative shadows on chest computed tomography scans among four the groups

In chest X-ray images, ground-glass opacities (GGO) and infiltrated shadows were significantly more frequent in Group 3 and 4 patients than in Group 1 patients (all *p* < 0.01), whereas these were less frequent in Group 2 patients than in Group 1 patients (*p* < 0.01). Additionally, GGO and infiltrated shadows in chest CT scans were more frequent in Group 3 and 4 patients than in Group 1 patients (Fig. 2).

### Treatment of the patients in the four groups

The summary of the therapeutic agents (remdesivir, antibiotics, steroids, tocilizumab, baricitinib, and anti-coagulant drugs) used in each group during the hospital stay is presented in Table 2. The patients in Group 2 were administered drugs less frequently than those in Group 1, with the exception of tocilizumab and baricitinib, whereas the patients in Group 3 received all drugs more frequently than those in Group 1. The patients in Group 4 were administered therapeutic agents, except antibiotics and baricitinib, more frequently than those in Group 1.

**Table 2:** Treatment of each respiratory symptoms group

	All (n = 3314)	Group 1 (n = 605)	Group 2 (n = 331)	Group 3 (n = 1229)	Group 4 (n = 1149)	*p* value
Remdesivir, %	35.8	24	16.4	46.9	35.9	< 0.0001^a = **/b = **/c = **^
Antibiotics, %	23.2	20.3	12.7	30.6	20.1	< 0.0001^a = **/b = **^
Steroids, %	50.4	38.1	24.6	64.7	49.2	< 0.0001^a = **/b = **/c = **^
Tocilizumab, %	9.8	5	3.3	15.2	8.5	< 0.0001^b = **/c = **^
Baricitinib, %	5.2	31.4	14.3	53.6	42.9	0.0014^b = *^
Anti-coagulant drugs, %	29.3	21	14	41.3	25.4	< 0.0001^a = **/b = **/c = *^

Data are shown as mean ± standard deviation

^a^Comparison of patients in group 1 versus group 2

^b^Comparison of patients in group 1 versus group 3

^c^Comparison of patients in group 1 versus group 4

*p < 0.05 **p < 0.01

### Impact of respiratory symptoms on clinical outcomes

**Fig. 3:**
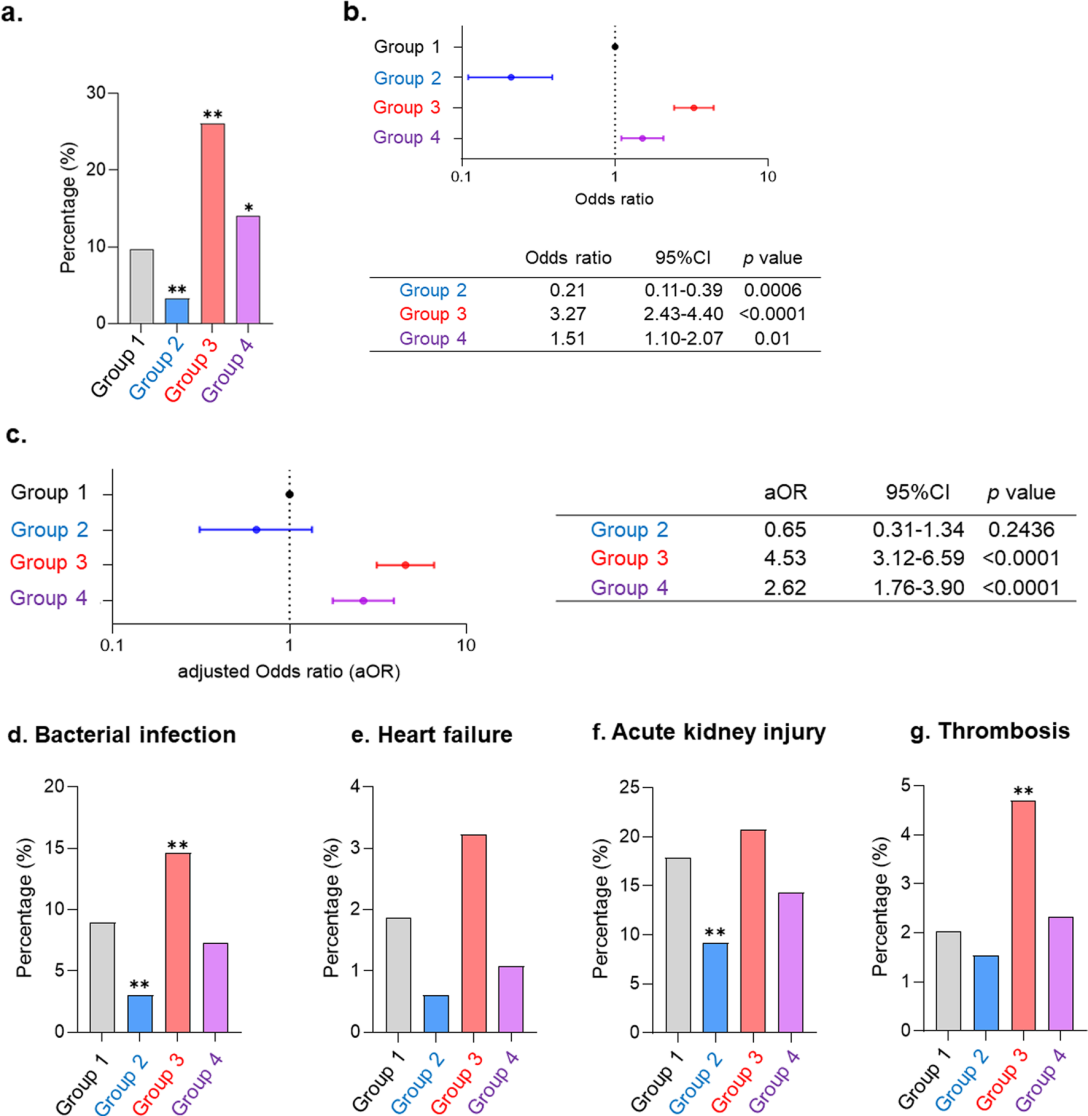
Relevance to clinical prognosis and percentage of complications in each group during in-patient treatment of COVID-19. **a** Univariate analysis of the proportion of poor prognosis cases in each group. **b** Comparison of odds ratios for poor clinical outcomes in each group. **c** Multivariate logistic regression analysis of the relationship between respiratory symptoms and critical outcomes in the whole cohort (adjusted for age, sex, BMI, smoking history and comorbidities (hypertension, cardiovascular disease, chronic kidney disease and chronic liver disease). **d** Univariate analysis of the proportion of patients with bacterial infection in each group. **e** Univariate analysis of the proportion of patients with heart failure in each group. **f** Univariate analysis of the proportion of patients with acute kidney injury in each group. **g** Univariate analysis of the proportion of patients with thrombosis in each group

Poor clinical outcomes (using high-flow oxygen therapy, invasive mechanical ventilation (IMV), ECMO, or death) were observed in 321 cases (9.8%) in Group 1, 11 cases (3.3%) in Group 2, 321 cases (26.1%) in Group 3, and 161 cases (14.0%) in Group 4 (Fig. 3a). Compared to Group 1 in univariate analysis, Group 2 had a significantly lower severity rate, and Groups 3 and 4 had a significantly higher severity rate. While Group 2 was associated with a better prognosis [OR (95% CI) = 0.21 (0.11–0.39)], Group 3 and 4 patients had the highest risk for severe disease [OR (95% CI) = 3.27 (2.43–4.40) and 1.51 (1.10–2.07)] (Fig. 3b). However, in the multivariate logistic regression analysis, which adjusted for patient characteristics and comorbidities, the significant difference between the prognosis of Group 1 and Group 2 disappeared [aOR = 0.65 (0.31–1.34)]. In contrast, Groups 3 and 4 remained significantly associated with a poor prognosis in the multivariate analysis [aOR (95% CI) = 4.53 (3.12–6.59) and 2.62 (1.76–3.90)] (Fig. 3c). In univariate analysis of treatments among the four groups, percentage of high-flow oxygen therapy was significantly lower in Group 2 and significantly higher in Groups 3 and 4 as compared to that in Group 1. Group 2 patients had a significantly lower rate of IMV use, and Group 3 patients were associated with increased rates of IMV and ECMO use as compared to those in Group 1. In a similar analysis, no significant differences were found for mortality among the four groups (Additional file 1: Fig. S2). Analysis of complications showed that percentages of bacterial infections were lower in Group 2 and higher in Group 3, when compared to Group 1. Moreover, Group 2 patients had a significantly lower frequency of acute kidney injury, and Group 3 patients had a significantly higher incidence of thrombosis than Group 1 patients (Fig. 3d–g).

### Relevance of clinical outcomes and respiratory symptoms

**Fig. 4:**
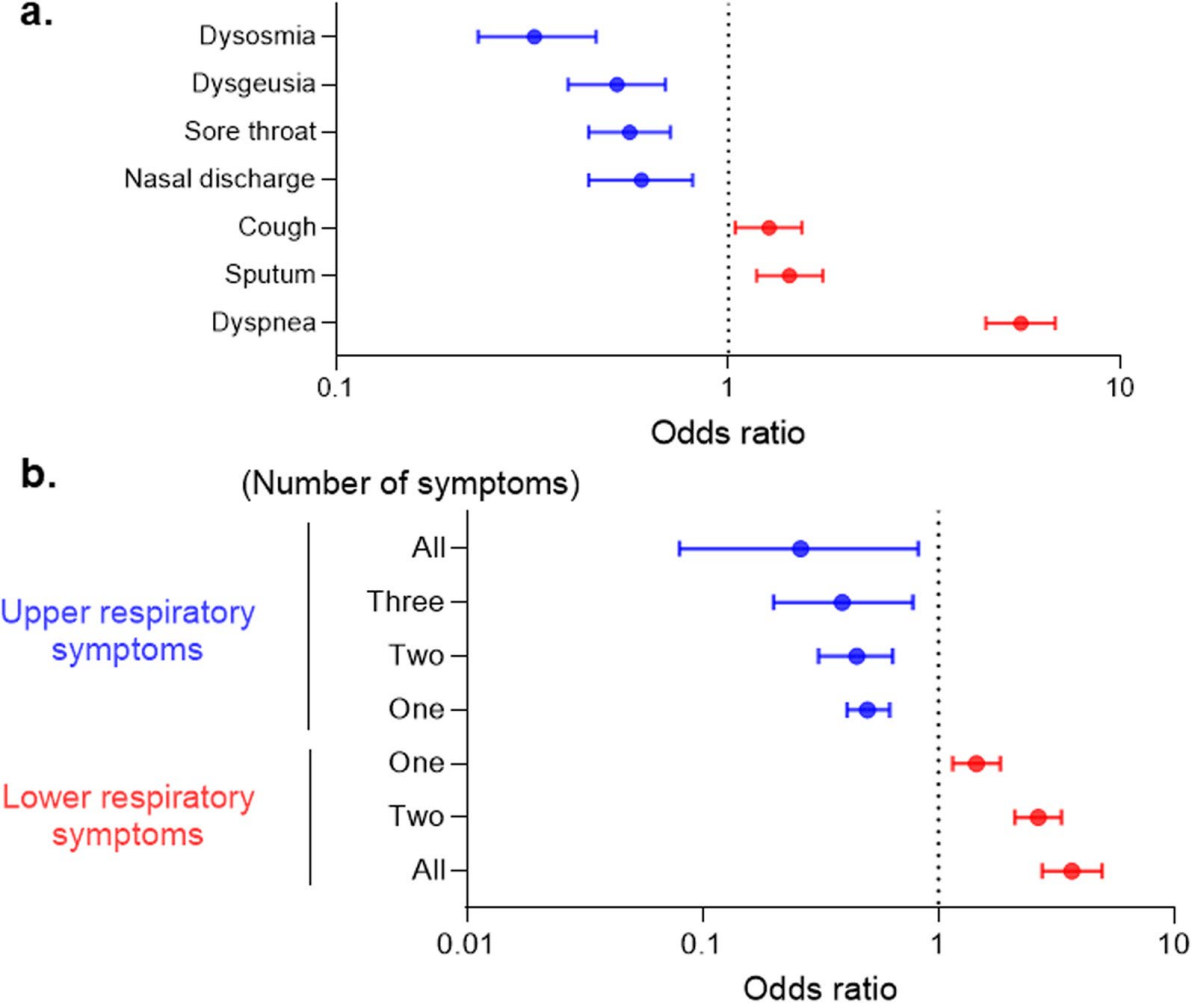
Relevance of symptoms in clinical prognosis. **a** Forest plot of odds ratios associated with poor clinical prognosis among respiratory symptoms. **b** Forest plot of odds ratios associated with poor clinical prognosis among numbers of respiratory symptoms

In the univariate analysis of the association of respiratory symptoms and clinical prognosis, all of the upper respiratory symptoms were good prognostic factors, whereas all of the lower respiratory symptoms were poor prognostic factors. Dysosmia (OR = 0.32 [0.23–0.46]) was the most favorable clinical factor, and dyspnea (OR = 5.56 [4.53–6.81]) was the worst risk factor associated with poor outcomes among respiratory symptoms. Interestingly, as the number of upper respiratory symptoms increased, the clinical course became better. Conversely, a higher incidence of lower respiratory symptoms was associated with a poorer prognosis (Fig. 4).

## Discussion

This is a large-scale study of the association between the clinical outcomes of COVID-19 and respiratory symptoms. In this study, we characterized the clinical course of patients and classified them into four groups based on their respiratory symptoms. We report that upper respiratory symptoms are favorable prognostic factors, and lower respiratory symptoms are poor factors, similarly to previous studies [2, 13, 15, 17]. Additionally, our study provided two novel findings with clinical relevance. First, we identified that patients with only lower respiratory symptoms had a worse clinical course than those with both upper and lower respiratory symptoms. Second, this study is the first to show the association between the numbers and types of respiratory symptoms and clinical outcomes in COVID-19 patients. This study suggests better clinical outcomes with upper respiratory symptoms and worse clinical outcomes with lower respiratory symptoms. This study demonstrated the importance of conducting a respiratory symptoms interview in the primary care of COVID-19.

The appearance of symptoms in COVID-19 is known to be influenced by age and sex, with otorhinolaryngological symptoms being more common in younger patients; systemic symptoms such as fever, malaise, and anorexia being more common in older patients; and dysosmia, headache, nasal obstruction, and fatigue more common in women [31]. Moreover, several previous studies have revealed that upper respiratory symptoms are associated with favorable clinical outcomes [13, 15]. Similar to previous studies, our study suggested that all upper respiratory symptoms were favorable prognostic factors. Upper respiratory symptoms have been reported to be highly common in mild outpatient cases of COVID-19 [32, 33]. Additionally, in the Delta and Omicron variants, mild upper respiratory symptoms, for instance nasal discharge and sneezing, appear more frequently [34]. In our study, upper respiratory symptoms were less frequent than those in the previous studies [32–34]. This is because this study focused on hospitalized patients and excluded the period after November 2021, when infections with Omicron variants were common. Moreover, this study suggested that olfactory dysfunction was associated with the best prognosis among upper respiratory symptoms. In summary, conducting a medical interview of patients with respect to their respiratory symptoms can be useful for clinicians in primary care to predict the disease severity.

Among the lower respiratory symptoms of COVID-19 evaluated in this study, cough occurred most frequently, and all symptoms of the lower respiratory tract were associated with a severe clinical prognosis, similar to the results of previous studies [2, 4, 17, 18]. There are two reasons for this. First, patients in Groups 3 and 4 showed GGO and infiltrated shadows on chest X-ray images and CT scans more frequently. Several studies have shown a relationship between the extent of pneumonia on chest X-ray images or CT scans and the clinical prognosis [35–39]. Lower respiratory symptoms were considered to reflect the presence of pneumonia as a poor prognostic factor. It would be useful to predict pneumonia based on only medical interviews of the respiratory symptoms observed. Second, patients in Group 3 had bacterial infections and embolism more frequently than those in other groups. Both bacterial infections and embolism with COVID-19 were reported to worsen clinical outcomes [40–43], and these complications may affect the prognosis of patients with lower respiratory symptoms alone. Additionally, patients in Groups 2 and 4 had a significantly lower incidence of comorbidities associated with severe outcomes such as diabetes, hypertension, and cardiovascular disease. However, patients in Group 3, with the worst prognosis, had a significantly different incidence of comorbidities associated with severe outcomes, except sex and BMI, as compared to that of Group 1 patients. As Fig. 3c shows, lower respiratory symptoms were a poor prognostic factor, independent of comorbidities associated with poor clinical outcome, and lower respiratory symptoms were useful markers in predicting the severity of COVID-19. In contrast, our multivariate analysis showed that upper respiratory symptoms were not an independent risk factor for poor outcomes in patients with COVID-19, and the better prognosis in Group 2 may have resulted from the lower incidence of comorbidities associated with severe disease, as suggested by previous studies [12, 13].

In the laboratory data, NLR, AST, ALT, and ferritin levels associated with poor clinical outcomes were significantly elevated in patients in Group 2, consistent with previous reports [44–47]. In addition, Alb, LDH, and HbA1c values improved in Group 2 patients with good prognosis, whereas they worsened in Group 3 patients with poor prognosis. [47, 48]

Several studies have confirmed that SARS-CoV-2 uses human ACE2 as a receptor to enter host cells [49, 50]. These proteins were highly expressed in the nasal epithelium, and their levels were downregulated throughout the lower respiratory tract and type II alveolar cells in the lung [51]. Thus, SARS-CoV-2 may enter through the nasal epithelium followed by entry into the lungs by inhalation, triggering pneumonia. Additionally, elevated ACE2 expression is expected to increase the viral load. Some studies have shown that a high viral load is associated with death and disease severity [52, 53]; thus, increased expression of ACE2 could lead to poor prognosis. Moreover, ACE2 expression is higher in the lungs of males than of females as shown by single cell RNA-seq [54]. Thus, the significantly higher proportion of males in Group 3 with poor prognosis may have been due to a higher ACE2 expression. In addition, obesity and smoking significantly increase ACE2 expression in the lungs and bronchial epithelium [55, 56]; this could further explain the higher rates of obesity and smoking in Groups 3 and 4 with lower respiratory symptoms. The lower frequency of these comorbidities in Groups 2 and 4 may be because SARS-CoV-2 accumulated and proliferated in the upper respiratory tract, where ACE2 expression was the highest. Interestingly, patients in Group 3 had a worse prognosis than patients in Group 4. Thus, elevated ACE2 expression in the lower respiratory tract could prevent the restriction of SARS-CoV-2 to the upper respiratory tract, resulting in poor prognosis. However, the association of ACE2 expression and COVID-19 severity has not been reported [57], and various complex factors are assumed to be involved in the severity of COVID-19.

Our study has several limitations. First, this study included only hospitalized patients with COVID-19, which might have resulted in a biased sample due to the high severity of the disease. Patients with only upper respiratory symptoms were often treated as mild cases. Therefore, the population in Group 2 may not adequately reflect the clinical characteristics of COVID-19 patients with only upper respiratory symptoms. Second, several previous studies used objective scoring tools to assess olfactory and taste disorders [58, 59], but this study included information from only medical interviews, which may be less accurate for symptoms. Additionally, in COVID-19, there are some cases of rapid and severe respiratory failure without dyspnea characterized by silent hypoxia [60]. Although the prognosis of asymptomatic cases was relatively better, clinicians should not solely rely on interviews and use biomarkers such as those measured using pulse oximetry. Further studies are needed to address these limitations and develop optimal treatment strategies in the near future.

## Conclusions

Based on the stratification of respiratory symptoms into upper and lower respiratory symptoms using medical interviews, clinicians may be able to predict the presence of pneumonia, clinical course, and complications of COVID-19. Especially in primary care, this easily obtained information is considered an important clinical tool.

## Supplementary Information

**Additional file 1. Supplemental Figure 1.** Study flow chart of patient identification and selectionStudy flow chart of patient identification and selection. A total of 117 records were excluded from the 3431 cases registered in the coronavirus disease 2019 (COVID-19) taskforce database owing to lack of essential clinical information. Ultimately, 3314 patients met the eligibility criteria, of which 2709 had respiratory symptoms. **Supplemental Figure 2.** Frequency of assisted respiration therapy and death in all four groups (**a**) Univariate analysis of the proportion of high-flow oxygen therapy with COVID-19 in each group. (**b**) Univariate analysis of the proportion of use of invasive mechanical ventilation (IMV) with COVID-19 in each group. (**c**) Univariate analysis of the proportion of use of extracorporeal membrane oxygenation (ECMO) with COVID-19 in each group. (**d**) Univariate analysis of the proportion of death with COVID-19 in each group. **Supplemental Table 1.**　Common non-respiratory symptoms in each group.

## Data Availability

The datasets generated during and/or analyzed during the current study available from the corresponding author on reasonable request.

## References

[1] Centers for Disease Control and Prevention. Interim clinical guidance for management of patients with confirmed coronavirus disease (COVID-19). https://stacks.cdc.gov/view/cdc/89980. Accessed 2 October 2022.

[2] Huang C, Wang Y, Li X, Ren L, Zhao J, Hu Y (2020). Clinical features of patients infected with 2019 novel coronavirus in Wuhan. China Lancet.

[3] Spinato G, Fabbris C, Polesel J, Cazzador D, Borsetto D, Hopkins C (2020). Alterations in smell or taste in mildly symptomatic outpatients with SARS-CoV-2 infection. JAMA.

[4] Stokes EK, Zambrano LD, Anderson KN, Marder EP, Raz KM, El Burai FS (2020). Coronavirus disease 2019 case surveillance—United States, January 22–May 30, 2020. MMWR Morb Mortal Wkly Rep.

[5] Wynants L, Van Calster B, Collins GS, Riley RD, Heinze G, Schuit E (2020). Prediction models for diagnosis and prognosis of covid-19: systematic review and critical appraisal. BMJ.

[6] Lombardi Y, Azoyan L, Szychowiak P, Bellamine A, Lemaitre G, Bernaux M (2021). External validation of prognostic scores for COVID-19: a multicenter cohort study of patients hospitalized in Greater Paris University Hospitals. Intensive Care Med.

[7] Zayet S, Kadiane-Oussou NJ, Lepiller Q, Zahra H, Royer PY, Toko L (2020). Clinical features of COVID-19 and influenza: a comparative study on Nord Franche-Comte cluster. Microbes Infect.

[8] Printza A, Constantinidis J (2020). The role of self-reported smell and taste disorders in suspected COVID-19. Eur Arch Otorhinolaryngol.

[9] da Rosa Mesquita R, Francelino Silva Junior LC, Santos Santana FM, Farias de Oliveira T, Campos Alcântara R, Monteiro Arnozo G (2021). Clinical manifestations of COVID-19 in the general population: systematic review. Wien Klin Wochenschr.

[10] Rocke J, Hopkins C, Philpott C, Kumar N (2020). Is loss of sense of smell a diagnostic marker in COVID-19: a systematic review and meta-analysis. Clin Otolaryngol.

[11] Sungnak W, Huang N, Bécavin C, Berg M, Queen R, Litvinukova M (2020). SARS-CoV-2 entry factors are highly expressed in nasal epithelial cells together with innate immune genes. Nat Med.

[12] Paderno A, Schreiber A, Grammatica A, Raffetti E, Tomasoni M, Gualtieri T (2020). Smell and taste alterations in COVID-19: a cross-sectional analysis of different cohorts. Int Forum Allergy Rhinol.

[13] Husain Q, Kokinakos K, Kuo YH, Zaidi F, Houston S, Shargorodsky J (2021). Characteristics of COVID-19 smell and taste dysfunction in hospitalized patients. Am J Otolaryngol.

[14] Whitcroft KL, Hummel T (2020). Olfactory dysfunction in COVID-19: diagnosis and management. JAMA.

[15] Piu N, Isabella A, Airoldi C, Aleni C, Sarro A, Faggiano F (2022). Taste and smell disorders in COVID-19 patients at a local healthcare trust in Northern Italy: a cross-sectional study. Ann Ig.

[16] Mao L, Jin H, Wang M, Hu Y, Chen S, He Q (2020). Neurologic manifestations of hospitalized patients with coronavirus Disease 2019 in Wuhan. China JAMA Neurol.

[17] Chen N, Zhou M, Dong X, Qu J, Gong F, Han Y (2020). Epidemiological and clinical characteristics of 99 cases of 2019 novel coronavirus pneumonia in Wuhan, China: a descriptive study. Lancet.

[18] Guan WJ, Ni ZY, Hu Y, Liang WH, Ou CQ, He JX (2020). Clinical characteristics of coronavirus disease 2019 in China. N Engl J Med.

[19] Husain M, Valayer S, Poey N, Rondinaud E, d'Humières C, Visseaux B (2022). Pulmonary bacterial infections in adult patients hospitalized for COVID-19 in standard wards. Infect Dis Now.

[20] Li J, Song CL, Wang T, Ye YL, Du JR, Li SH (2021). Etiological and epidemiological characteristics of severe acute respiratory infection caused by multiple viruses and Mycoplasma pneumoniae in adult patients in Jinshan, Shanghai: a pilot hospital-based surveillance study. PLoS ONE.

[21] Namkoong H, Edahiro R, Fukunaga K, Shirai Y, Sonehara K, Tanaka H, et al. Japan COVID-19 Task Force: a nation-wide consortium to elucidate host genetics of COVID-19 pandemic in Japan. medRxiv. 2021:05.17.21256513.

[22] Tanaka H, Lee H, Morita A, Namkoong H, Chubachi S, Kabata H (2021). Clinical characteristics of patients with coronavirus disease (COVID-19): preliminary baseline report of Japan COVID-19 Task Force, a nationwide consortium to investigate host genetics of COVID-19. Int J Infect Dis.

[23] COVID-19 therapeutic trial synopsis; 2022. https://www.who.int/publications/i/item/covid-19-therapeutic-trial-synopsis. Accessed 3 June 2022.

[24] O’Driscoll M, Ribeiro dos Santos G, Wang L, Cummings DAT, Azman AS, Paireau J (2021). Age-specific mortality and immunity patterns of SARS-CoV-2. Nature.

[25] Geng L, He C, Kan H, Zhang K, Mao A, Zhang C (2021). The association between blood pressure levels and mortality in critically ill patients with COVID-19 in Wuhan, China: a case-series report. Hypertens Res.

[26] Huang I, Lim MA, Pranata R (2020). Diabetes mellitus is associated with increased mortality and severity of disease in COVID-19 pneumonia—a systematic review, meta-analysis, and meta-regression. Diabetes Metab Syndr.

[27] Santoso A, Pranata R, Wibowo A, Al-Farabi MJ, Huang I, Antariksa B (2021). Cardiac injury is associated with mortality and critically ill pneumonia in COVID-19: a meta-analysis. Am J Emerg Med.

[28] Singh J, Malik P, Patel N, Pothuru S, Israni A, Chakinala RC (2022). Kidney disease and COVID-19 disease severity-systematic review and meta-analysis. Clin Exp Med.

[29] Jin JM, Bai P, He W, Wu F, Liu XF, Han DM (2020). Gender differences in patients with COVID-19: focus on severity and mortality. Front Public Health.

[30] Hendren NS, de Lemos JA, Ayers C, Das SR, Rao A, Carter S (2021). Association of body mass index and age with morbidity and mortality in patients hospitalized with COVID-19: results from the American Heart Association COVID-19 cardiovascular Disease Registry. Circulation.

[31] Lechien JR, Chiesa-Estomba CM, Place S, Van Laethem Y, Cabaraux P, Mat Q (2020). Clinical and epidemiological characteristics of 1420 European patients with mild-to-moderate coronavirus disease 2019. J Intern Med.

[32] Tenforde MW, Billig Rose E, Lindsell CJ, Shapiro NI, Files DC, Gibbs KW (2020). Characteristics of adult outpatients and inpatients with COVID-19— 11 Academic Medical Centers, United States, March-May 2020. MMWR Morb Mortal Wkly Rep.

[33] Killerby ME, Link-Gelles R, Haight SC, Schrodt CA, England L, Gomes DJ (2020). Characteristics associated with hospitalization among patients with COVID-19—Metropolitan Atlanta, Georgia, March-April 2020. MMWR Morb Mortal Wkly Rep.

[34] Menni C, Valdes AM, Polidori L, Antonelli M, Penamakuri S, Nogal A (2022). Symptom prevalence, duration, and risk of hospital admission in individuals infected with SARS-CoV-2 during periods of omicron and delta variant dominance: a prospective observational study from the ZOE COVID Study. Lancet.

[35] Shen B, Hoshmand-Kochi M, Abbasi A, Glass S, Jiang Z, Singer AJ (2021). Initial chest radiograph scores inform COVID-19 status, intensive care unit admission and need for mechanical ventilation. Clin Radiol.

[36] Homayounieh F, Zhang EW, Babaei R, Karimi Mobin H, Sharifian M, Mohseni I (2020). Clinical and imaging features predict mortality in COVID-19 infection in Iran. PLoS ONE.

[37] Zheng Y, Wang L, Ben S (2021). Meta-analysis of chest CT features of patients with COVID-19 pneumonia. J Med Virol.

[38] Li K, Wu J, Wu F, Guo D, Chen L, Fang Z (2020). The clinical and chest CT features associated with severe and critical COVID-19 pneumonia. Invest Radiol.

[39] Colombi D, Bodini FC, Petrini M, Maffi G, Morelli N, Milanese G (2020). Well-aerated lung on admitting chest CT to predict adverse outcome in COVID-19 pneumonia. Radiology.

[40] Garcia-Vidal C, Sanjuan G, Moreno-García E, Puerta-Alcalde P, Garcia-Pouton N, Chumbita M (2021). Incidence of co-infections and superinfections in hospitalized patients with COVID-19: a retrospective cohort study. Clin Microbiol Infect.

[41] Musuuza JS, Watson L, Parmasad V, Putman-Buehler N, Christensen L, Safdar N (2021). Prevalence and outcomes of co-infection and superinfection with SARS-CoV-2 and other pathogens: a systematic review and meta-analysis. PLoS ONE.

[42] Li JY, Wang HF, Yin P, Li D, Wang DL, Peng P (2021). Clinical characteristics and risk factors for symptomatic venous thromboembolism in hospitalized COVID-19 patients: a multicenter retrospective study. J Thromb Haemost.

[43] Meena RA, Sharifpour M, Gaddh M, Cui X, Xie Y, Di M (2021). COVID-19-associated venous thromboembolism portends worse survival. Semin Vasc Surg.

[44] Ponti G, Maccaferri M, Ruini C, Tomasi A, Ozben T (2020). Biomarkers associated with COVID-19 disease progression. Crit Rev Clin Lab Sci.

[45] Malik P, Patel U, Mehta D, Patel N, Kelkar R, Akrmah M (2021). Biomarkers and outcomes of COVID-19 hospitalisations: systematic review and meta-analysis. BMJ Evid Based Med.

[46] Cheng L, Li H, Li L, Liu C, Yan S, Chen H (2020). Ferritin in the coronavirus disease 2019 (COVID-19): a systematic review and meta-analysis. J Clin Lab Anal.

[47] Zhu Z, Mao Y, Chen G (2021). Predictive value of HbA1c for in-hospital adverse prognosis in COVID-19: a systematic review and meta-analysis. Prim Care Diabetes.

[48] Dai Z, Zeng D, Cui D, Wang D, Feng Y, Shi Y (2020). Prediction of COVID-19 patients at high risk of progression to severe disease. Front Public Health.

[49] Hoffmann M, Kleine-Weber H, Schroeder S, Krüger N, Herrler T, Erichsen S (2020). SARS-CoV-2 cell entry depends on ACE2 and TMPRSS2 and is blocked by a clinically proven protease inhibitor. Cell.

[50] Bilinska K, Jakubowska P, Von Bartheld CS, Butowt R (2020). Expression of the SARS-CoV-2 entry proteins, ACE2 and TMPRSS2, in cells of the olfactory epithelium: identification of cell types and trends with age. ACS Chem Neurosci.

[51] Hou YJ, Okuda K, Edwards CE, Martinez DR, Asakura T, Dinnon KH (2020). SARS-CoV-2 Reverse genetics reveals a variable infection gradient in the respiratory tract. Cell.

[52] Pujadas E, Chaudhry F, McBride R, Richter F, Zhao S, Wajnberg A (2020). SARS-CoV-2 viral load predicts COVID-19 mortality. Lancet Respir Med.

[53] Aggarwal S, Aggarwal S, Aggarwal A, Jain K, Minhas S (2020). High viral load and poor ventilation: cause of high mortality from COVID-19. Asia Pac J Public Health.

[54] Zhao Y, Zhao Z, Wang Y, Zhou Y, Ma Y, Zuo W (2020). Single-Cell RNA expression profiling of ACE2, the receptor of SARS-CoV-2. Am J Respir Crit Care Med.

[55] Higham A, Singh D (2020). Increased ACE2 expression in bronchial epithelium of COPD patients who are overweight. Obesity (Silver Spring).

[56] Leung JM, Yang CX, Tam A, Shaipanich T, Hackett TL, Singhera GK (2020). ACE-2 expression in the small airway epithelia of smokers and COPD patients: implications for COVID-19. Eur Respir J.

[57] Hippisley-Cox J, Young D, Coupland C, Channon KM, Tan PS, Harrison DA (2020). Risk of severe COVID-19 disease with ACE inhibitors and angiotensin receptor blockers: cohort study including 8.3 million people. Heart.

[58] Moein ST, Hashemian SM, Mansourafshar B, Khorram-Tousi A, Tabarsi P, Doty RL (2020). Smell dysfunction: a biomarker for COVID-19. Int Forum Allergy Rhinol.

[59] Ninchritz-Becerra E, Soriano-Reixach MM, Mayo-Yánez M, Calvo-Henríquez C, Martínez-Ruiz de Apodaca P, Saga-Gutiérrez C (2021). Subjective evaluation of smell and taste dysfunction in patients with mild COVID-19 in Spain. Med Clin (Barc).

[60] Rahman A, Tabassum T, Araf Y, Al Nahid A, Ullah MA, Hosen MJ (2021). Silent hypoxia in COVID-19: pathomechanism and possible management strategy. Mol Biol Rep.

