# Supplementary material for: Impact of upper and lower respiratory symptoms on COVID-19 outcomes: a multicenter retrospective cohort study
Source: Respir Res. 2022 Nov 15;23:315. doi: 10.1186/s12931-022-02222-3 (PMC9665023; doi:10.1186/s12931-022-02222-3)
Supplement: Supplementary file 1 — Additional file 1. Supplemental Figure 1. Study flow chart of patient identification and selectionStudy flow chart of patient identification and selection. A total of 117 records were excluded from the 3431 cases registered in the coronavirus disease 2019 (COVID-19) taskforce database owing to lack of essential clinical information. Ultimately, 3314 patients met the eligibility criteria, of which 2709 had respiratory symptoms. Supplemental Figure 2. Frequency of assisted respiration therapy and death in all four groups (a) Univariate analysis of the proportion of high-flow oxygen therapy with COVID-19 in each group. (b) Univariate analysis of the proportion of use of invasive mechanical ventilation (IMV) with COVID-19 in each group. (c) Univariate analysis of the proportion of use of extracorporeal membrane oxygenation (ECMO) with COVID-19 in each group. (d) Univariate analysis of the proportion of death with COVID-19 in each group. Supplemental Table 1. Common non-respiratory symptoms in each group. [file 12931_2022_2222_MOESM1_ESM.docx]

**SUPPORTING INFORMATION**


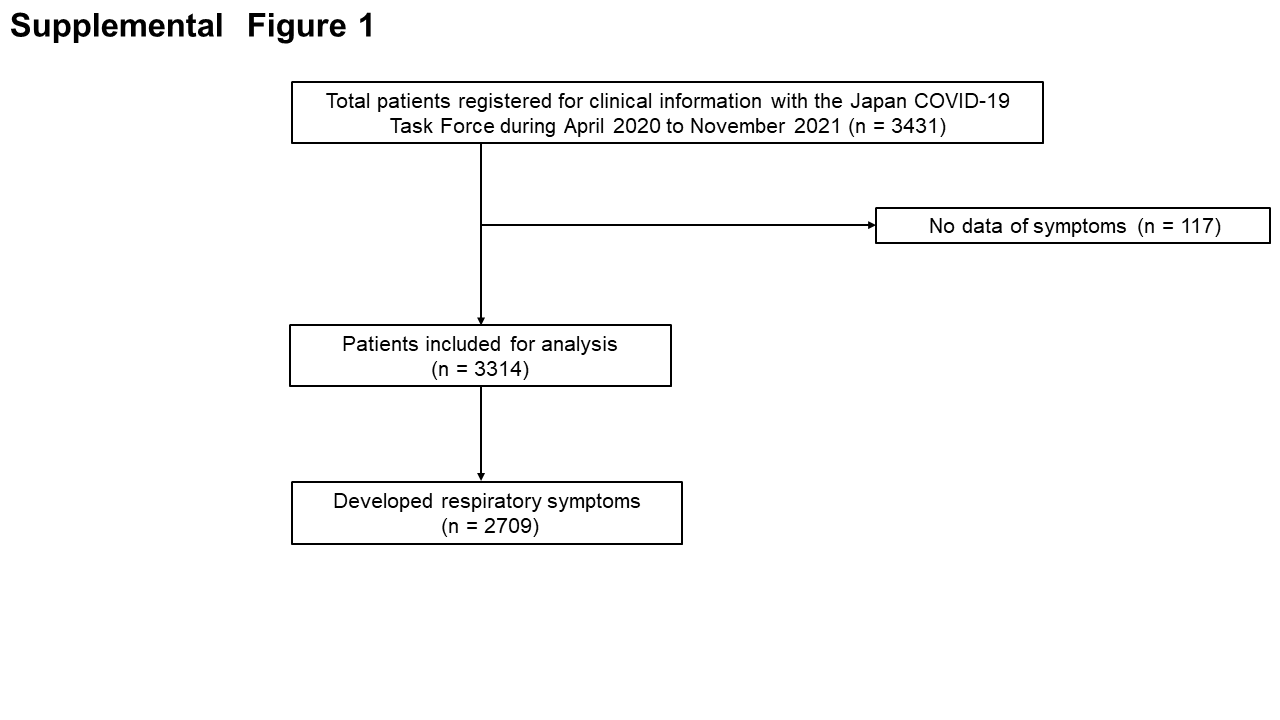


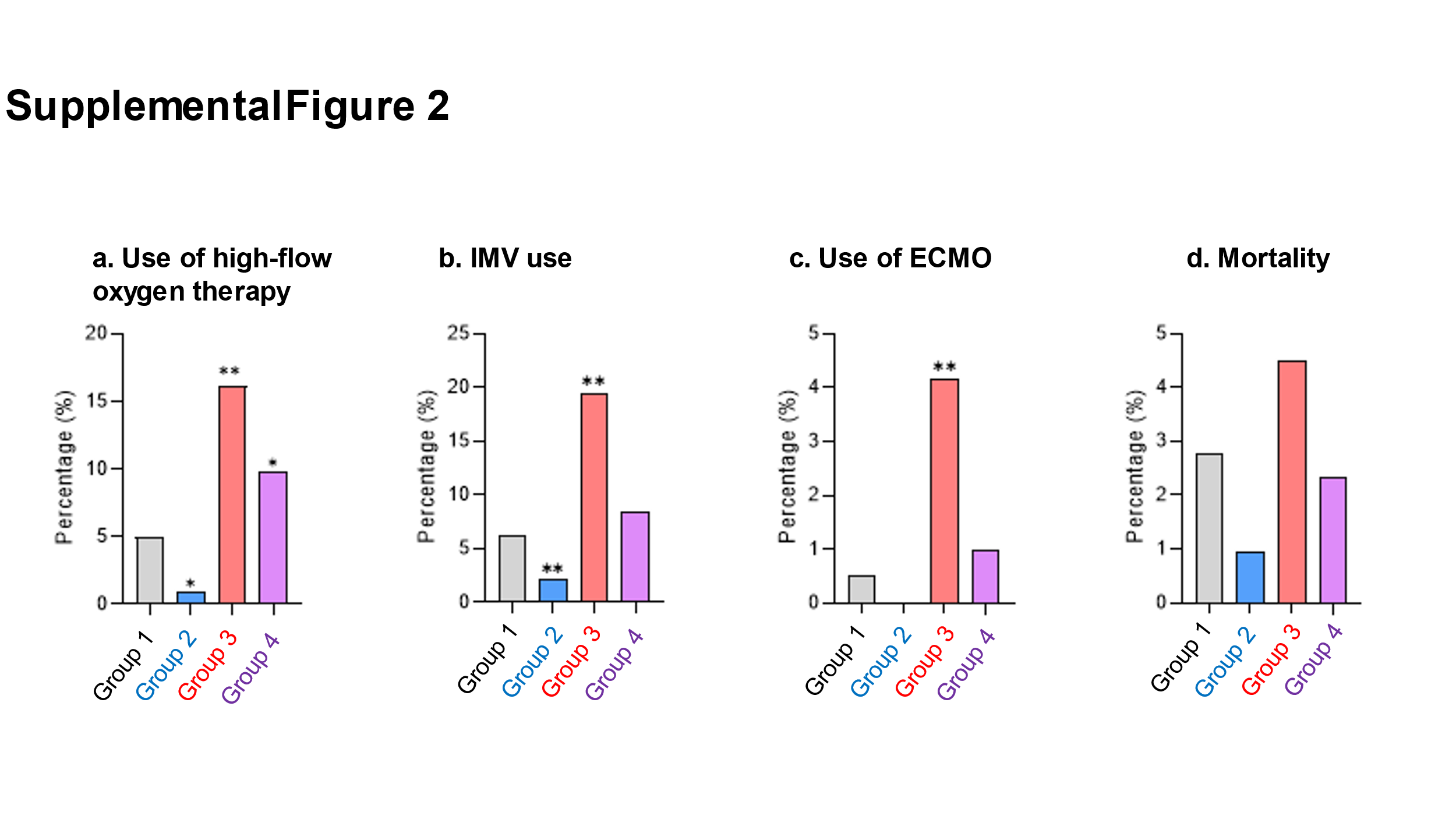


| **Supplemental Table 1　Common non-respiratory symptoms in each group** | | | | | | |
| --- | --- | --- | --- | --- | --- | --- |
|  | **All  (n = 3314)** | **Group 1 (n = 605)** | **Group 2 (n = 331)** | **Group 3 (n = 1229)** | **Group 4 (n = 1149)** | ***p* value** |
| **Fever, %** | **80.8** | **72.3** | **71.5** | **81.7** | **86.7** | **< 0.0001^b = **/c = **^** |
| **Fatigue, %** | **54.9** | **38.8** | **41.7** | **58.5** | **63** | **< 0.0001^b = **/c = **^** |
| **Diarrhea, %** | **17.4** | **10.8** | **16.2** | **12.4** | **26.4** | **< 0.0001^a = */c = **^** |
| **Nausea, %** | **9.2** | **6.4** | **8.3** | **7** | **13.1** | **< 0.0001^c = **^** |
| **Consciousness disturbance, %** | **3.7** | **4.2** | **0** | **5.6** | **2** | **< 0.0001^a = **/c = *^** |
| **Abdominal pain, %** | **3.5** | **2** | **2.1** | **2.6** | **5.1** | **0.0004^c = **^** |
| **Bloody stool, %** | **0.5** | **0.7** | **0** | **0.6** | **0.4** | **0.4381** |
| Data are shown as mean ± standard deviation (SD). a Comparison of patients in group 1 versus group 2 b Comparison of patients in group 1 versus group 3 c Comparison of patients in group 1 versus group 4 * *p* < 0.05 ** *p* < 0.01 | | | | | | |
